# VERB™ — A Social Marketing Campaign to Increase Physical Activity Among Youth

**Published:** 2004-06-15

**Authors:** Faye Wong, Marian Huhman, Lori Asbury, Rosemary Bretthauer-Mueller, Susan McCarthy, Paula Londe, Carrie Heitzler

**Affiliations:** VERB Campaign, Division of Adolescent and School Health (DASH), National Center for Chronic Disease Prevention and Health Promotion (NCCDPHP), Centers for Disease Control and Prevention; VERB Campaign, DASH, NCCDPHP, CDC, Atlanta, Ga; VERB Campaign, DASH, NCCDPHP, CDC, Atlanta, Ga; VERB Campaign, DASH, NCCDPHP, CDC, Atlanta, Ga; VERB Campaign, DASH, NCCDPHP, CDC, Atlanta, Ga; VERB Campaign, DASH, NCCDPHP, CDC, Atlanta, Ga; VERB Campaign, Division of Nutrition and Physical Activity, NCCDPHP, CDC, Atlanta, Ga

## Abstract

The VERB campaign is a multiethnic media campaign with a goal to increase and maintain physical activity among *tweens*, or children aged nine to 13 years. Parents, especially mothers aged 29 to 46, and other sources of influence on tweens (e.g., teachers, youth program leaders) are the secondary audiences of the VERB initiative. VERB applies sophisticated commercial marketing techniques to address the public health problem of sedentary lifestyles of American children, using the social marketing principles of product, price, place, and promotion. In this paper, we describe how these four principles were applied to formulate the strategies and tactics of the VERB campaign, and we provide examples of the multimedia materials (e.g., posters, print advertising, television, radio spots) that were created.

## Introduction

In response to increased concern about the health of our nation’s youth, Congress appropriated $125 million in 2001 to the Centers for Disease Control and Prevention (CDC) to develop a national media campaign to change children’s health behaviors. The CDC’s response to this broad mandate was to focus on the sedentary lifestyle of young adolescents and to develop VERB™, a multiethnic campaign launched in June 2002 to increase and maintain physical activity among tweens, or children aged nine to 13 years. These children are between childhood and adolescence and are beginning to make their own lifestyle decisions. Parents, especially mothers aged 29 to 46, and other sources of influence on tweens (e.g., teachers, youth program leaders) provide the secondary audiences for the VERB initiative.

During the past 20 years, the combination of decreased physical activity and unhealthful eating has resulted in a doubling of the percentage of overweight children and adolescents (1). Recent reports indicate that five of every eight children aged nine to 13 do not participate in any organized physical activity during their non-school hours, and almost one fourth do not engage in any free-time physical activity (2). More than one in seven children aged six to 19 years are overweight (3), and type 2 diabetes, a disease traditionally restricted to adults, has been reported among adolescents (4).

## A Social Marketing Framework

VERB uses a social marketing framework that applies sophisticated commercial marketing techniques to address the public health problem of sedentary lifestyles among American children. The first year of the campaign consisted of national advertising, plus extra marketing activities in nine CDC-selected communities. The CDC based its selection of the nine communities on a variety of factors, including the size of the media market, racial and ethnic diversity, geographic diversity across the United States, existing infrastructure, and population size. Six of the nine communities received even more local advertising so that the CDC could evaluate whether the added media made a measurable difference in behavioral outcomes. These six evaluated communities were called “high-dose” communities.

**Figure:**
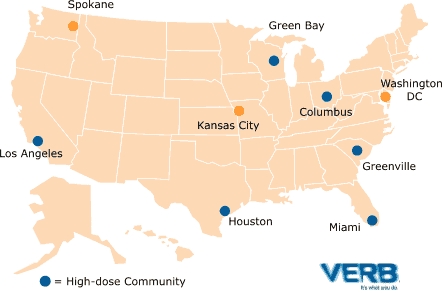
Nine Communities Receiving Extra Marketing Activities

Before campaign planners launched or named the campaign, they conducted extensive research on tweens and parents to gain an understanding of their attitudes, beliefs, and behaviors related to participation in physical activity. Research included numerous in-person focus groups, interviews, and ethnographic inquiries among multiethnic groups across the country. Additional audience research was conducted separately with African American, Hispanic/Latino, American Indian, and Asian American tweens and parents to gain deeper insights into their physical activity views and practices. The understandings gained contributed to the creation of VERB, a “for tweens, by tweens” brand that embraces the characteristics valued by tweens. VERB is not an acronym but is the word *verb* as a part of speech, meaning an action word. The tag line is, “It’s what you do.” (Formative research reports for the VERB campaign are available from http://www.cdc.gov/youthcampaign/research/resources.htm.)

Campaign planners applied the four *Ps* of commercial marketing — product, price, place, and promotion (5) — along with findings from the audience research to develop VERB as a social marketing campaign. In this paper, we describe how the four *Ps* helped to formulate the strategies and tactics of the VERB campaign.

### Product

In social marketing, *product* is the desired behavior for the targeted audience. The VERB campaign’s product is physical activity — a voluntary action that requires personal choice and internal motivation if it is to be performed repeatedly. The VERB “sales package” includes the intangible benefits of physical activity (e.g., enjoyment) in addition to tangible objects or services that support behavior change (6).

For tweens, choosing to be physically active means giving up something they may like doing better. In today’s world, numerous activities compete for tweens’ attention, and tweens and their parents must overcome many barriers to become physically active, including lack of transportation, safety concerns, cost, and perceived lack of time (2). Children report spending more than 4.5 hours daily watching television, playing video games, or using the computer; parents report their children’s screen time to be almost 6.5 hours daily (7). The VERB campaign’s goal is to win or gain a greater market share of time tweens spend on sedentary activities.

The campaign’s strategy is to influence participation in physical activity by associating it with the benefits that tweens value, such as spending time with friends, playing, having fun, having an opportunity to be active with parents, and gaining recognition from peers and adults. In addition, VERB offers tweens something else they value: the opportunity to explore and discover the world around them. Early VERB advertising stimulated curiosity about the brand and enticed tweens to identify and try the activities or VERBs (e.g., swim, run, jump) that most appealed to them.

### Price

*Price* represents a balance of product benefits and costs to a consumer. When contemplating the purchase of a tangible product such as a tennis racquet, for example, a consumer balances the potential benefits of playing tennis against the price tag (5,6). When the product is behavior change, the concept still holds: what are the benefits and costs of changing behavior (8)? For physical activity, the costs can be financial (e.g., price of dance classes), psychological (e.g., the tween does not “feel good enough” to participate in physical activity or organized sports), environmental (e.g., the neighborhood does not have sidewalks), or related to time (e.g., both parents work, leaving tweens with no time for supervised physical activity). VERB messages are designed to convince tweens and their parents that physical activity has the “right price” — that benefits outweigh costs.

Audience research yielded numerous insights into how tweens and parents view the benefits of physical activity; the CDC thus had information to show how the benefits of physical activity exceed those of non-active pursuits. The VERB campaign weaves these benefits throughout its messages, strategies, and tactics to make physical activity appealing and inviting to tweens.

### Place

**Figure:**
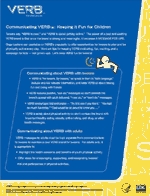
Download the VERB Coolness Tip Sheet (PDF 313K)

*Place* is where the target audience either performs the behavior or accesses programs or services; place must be readily available to enable the desired action (5,6). A VERB place is where tweens can be physically active in a safe environment. As interest or demand rises, parents and communities need to increase the supply of accessible places and opportunities for tweens to be active every day. If the supply side is inadequate or inaccessible, tweens cannot act on their interest and intent to be more active. For VERB, a place may be a backyard, youth-serving organization, community-based organization, church, park or recreation department, school, public or private sports organization, business, government agency, or any other place that can provide facilities and year-round or periodic event-based opportunities for tweens to be physically active and have fun.

Partner organizations such as parks, schools, and youth-serving organizations can reap the benefits of tweens’ affinity for the VERB brand and interest in becoming more active. Keeping VERB a “cool brand for tweens” is a critically important goal for partners as they collaborate on the campaign. (More information on keeping VERB “cool” is available from the VERB Coolness Tip Sheet.)

### Promotion

*Promotion* is not simply the placement of advertisements — communication messages and activities are included as well, and those in charge of promotions must consider multiple ways to reach the target audience to promote the benefits of the behavior change, including its product, price, and place components (6,7). The following sections provide descriptions and examples of the VERB campaign’s messaging strategies, advertising and marketing strategies, and campaign tactics for reaching tweens and parents.

#### Messaging strategies

Advertising and promotions do more than merely sell the features of a product; they depict a lifestyle that consumers aspire to achieve. By association, consumers perceive the product as providing the means to a desired outcome. In commercials, for example, a soft drink is more than a drink; it is a social experience. Running shoes are more than footwear; they make a statement about an individual’s lifestyle. In the VERB campaign, commercial strategies for marketing to youth are applied to public health and used to “sell” physical activity to tweens, creating a distinct brand culture for VERB.

VERB aims to sell physical activity to consumers, but boys and girls cannot simply go to a store to buy it. Rather, tweens must develop a positive disposition to physical activity through a positive association and relationship with the VERB brand. To make the product of physical activity compelling and cool to tweens, VERB messages diverge from the “just-the-facts” delivery that is central to many public health campaigns. Rather than say, “Engage in moderate-to-vigorous physical activity for at least 60 minutes each day,” the campaign asks tweens to discover new activities they like to do.

The fact that the campaign’s brand name, VERB, immediately connotes action increases the audience’s comprehension of the brand. To inspire tweens to do their own VERBs (physical activities), the campaign does not simply *tell* tweens that physical activity is for all tweens, it *shows* them with appealing visuals. Casting for television and print ads includes children of varied racial and ethnic backgrounds, body weights, and ability levels — including children with disabilities — to convey a sense of “kids like me do this” and “I can do that.” VERB shows tweens playing backyard games in addition to participating in organized physical activities such as team sports.

If tweens are inspired by a VERB commercial and motivated to be active, parents will more likely support their participation. Had the campaign’s strategy primarily focused on asking parents to encourage their children to be active, however, tweens might not have embraced VERB as their own brand. The campaign would have taken on a parenting agenda rather than becoming something for tweens. Notably, VERB features positive “can do” messages, not negative adult-delivered or adult-enforced “must do” or “don’t do” messages.

Parents and others who influcence tweens are asked to support, recognize, and praise children for being active; to encourage them to try new activities; and to be physically active as a family or group. VERB gives tips on how to communicate to tweens and engage them in being active in creative, positive, and fun ways. At the same time, facts on the health risks of inactivity and excessive screen time and on the benefits of being physically active are messages for adults. The messages are further tailored for different audiences, especially ethnic audiences, to address the specific parenting priorities learned through audience research.

All VERB campaign messages and the way they are presented in ads (i.e., television, radio, print) are tested with tween-aged focus groups to ensure they are motivating, clearly understood, and resonating positively. The messages are tested primarily with mothers to ensure they are acceptable and not offensive or inappropriate.

#### Advertising and marketing strategies

**Figure:**
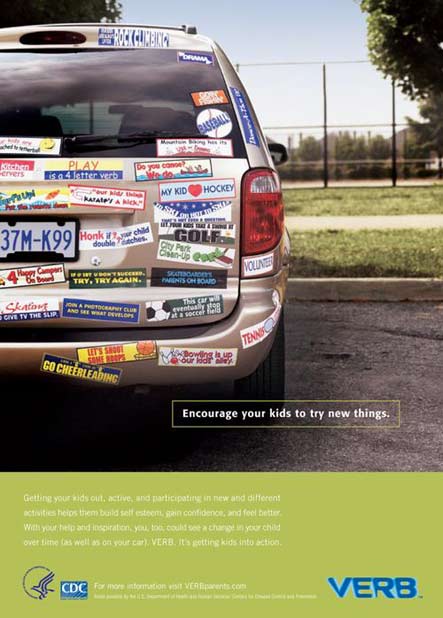
VERB™ Bumper sticker ad Poster Image: A car is covered with a humorously large number of bumper stickers, all of them describing the sports in which the owners' children participate. Poster Text: Encourage your kids to try new things. Getting your kids out, active, and participating in new and different activities helps them build self esteem, gain confidence, and feel better. With your help and inspiration, you, too, could see a change in your child over time (as well as your car). VERB. It's getting kids into action. Legal Text: For more information visit VERBparents.com HHS logo, CDC Logo, VERB logo

The VERB campaign strives for high brand awareness and affinity among tweens. The theory is that when tweens are positively bonded with VERB, they will be more receptive to messages about physical activity. In the first year of VERB, marketing efforts were dedicated to creating and introducing the VERB brand to tweens. As a previously nonexistent brand, VERB initially had no value to tweens. To sell VERB successfully to tweens as “their brand for having fun,” the campaign associates itself with popular kids’ brands, athletes, and celebrities, and activities and products that are cool, fun, and motivating.

Communications for parents and tweens are separated from each other to maintain tweens’ positive affinity for the VERB brand and to avoid associating physical activity with something adults say “they have to do.” Tweens’ interests and trends evolve rapidly, whether the subject is music, clothing, electronics, or the enjoyment of being active. It is all about being “cool” to their friends and doing what’s popular. The delicate balance of keeping the VERB brand cool for tweens while relying on parents and other adult influencers’ support to help them be active is an ongoing campaign challenge.

VERB is distinct from traditional public service announcement (PSA) campaigns because advertising placement is purchased. In the first year of VERB, the campaign averaged 115 weekly gross rating points^1^ (GRPs) in the national television media market with 50 percent more GRPs in the high-dose communities. The purchase of media enables the campaign to control when and where advertising appears and to concentrate ad placement in delivery channels that reach the most tweens; popular delivery channels include kids’ television networks (e.g., Nickelodeon, Cartoon Network) and teen magazines (e.g., *Teen People, Seventeen*). Furthermore, it assures that tweens are sufficiently exposed to the advertising to recognize and develop a personal relationship with the brand and to understand the campaign’s messages to become active and have fun doing it. Having the funding to purchase media placements allows VERB to compete with commercial youth marketers to capture the attention and brand loyalty of tweens.

#### Campaign tactics

VERB employs a broad mix of campaign tactics to reach tweens and their parents. The campaign is designed to surround tweens at home, in school, and in the community to give VERB visible presence in tweens’ everyday lives.

Campaign Materials

**Figure:**
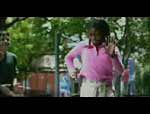
"Hip Hop Scotch" Watch "Hip Hop Scotch" VERB TV spot (RM 1.9mb)

30-second Television Spot
VISUALS Different shots all taking place at Mecca Park in Houston, Texas. The lead tween introduces herself as “Andrea” and explains how to play a game called “Hip-Hop Scotch.” Spot opens with a stop-motion shot of tweens dancing. Shots featured are close-ups of the kids, as well as tweens playing the game. A hip-hop beat accompanies the shots of children and the game, while the rules are explained with voice-over. Title graphics of key words and images appear throughout, such as “Hip Hop Scotch,” “Dance Moves,” and a diagram of the boxes. Additional shots include kids playing the game and dancing, and a stop-motion shot of girl dancing. The spot ends with an aerial shot of a group of kids looking up and saying the tagline, “VERB, It’s what you do,” as graphic animation depicting the logo and tagline fade in. The closing shot is a graphic with the word “VERB” being written with chalk on asphalt.
GIRL TWEEN Andrea: Hey, I’m Andrea and we play at Mecca Park in Houston, Texas. We’ve got lots of VERBs here. Some games we even make up. Here’s one we call Hip-Hop Scotch. (Title Graphic— “Andrea”/“Hip-Hop Scotch”) Andrea: It’s old school meets new school. You draw boxes, but instead of numbers you write in dance moves like spin, bounce, step, or shake. (Title Graphic— “Dance Moves”) Andrea: Wherever it lands, that’s your thing. If it lands in the question mark, do anything. (Title Graphic— “Do anything”) Andrea: That’s “Hip-Hop Scotch” in Houston. Every day is game day.
GIRL TWEEN Girl Tween 2 & 3: So get out there and go play! (Title Graphic— “Go Play”)

**Figure:**
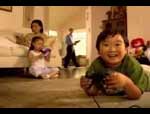
"Future" Watch "Future" VERB TV spot (RM 3.6mb)

60-second Television Spot
VISUALS A father in his mid-30s returns home from work feeling very tired. When he steps into his house, he sees his two children (aged ten and seven years) playing video games in the living room. At that point, he feels very proud and happy that he has provided a comfortable environment for his children. But when he glances through a picture of himself and his parents taken during his graduation, it triggers his memory of the old days, and then we see a flash-back of him. When he was a young child, his parents always took him out to play and supported him in all kinds of physical activities. All these activities helped him to become a healthy and well–balanced person. We then we see him back in today’s life. He decides that he shouldn’t give himself any more excuses and should be like his parents in helping his children to step into a brighter future. He takes out his baseball gloves and together with his wife, takes the children out to play. The voice-over reminds the audience that physical activities can help children do better in school, boost their confidence, and help develop both their body and mind. The spot ends with a call to action telling parents and adults that they need to encourage children to have 60 minutes of physical activity every day, as the words “VERB — A Campaign To Build Healthy Children” appear on the screen.
ADULT VOICE–OVER If you want your children to step into a brighter future, encourage them to participate in more physical activities. It could help them do better in school and boost their confidence for tomorrow’s challenges!
ADULT VOICE–OVER Girl Tween 2 & 3: So get out there and go play! (Title Graphic— “Go Play”)
SIGN–OFF VERB™ A campaign to build healthy children www.VERBparents.com
SIGN–OFF U.S. Department of Health and Human Services Centers for Disease Control and Prevention

**Figure:**
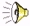
"Hip Hop Scotch" Hear "Hip Hop Scotch" VERB radio spot

30-second Radio Spot
VOICE-OVER Hey, check out these kids who made up their own VERB…
VOICEOVER ANDREA: Hey, I'm Andrea and we play in Houston, Texas. We've got lots of VERBs here, some games we even make up. Here's one we call "Hip-Hop Scotch." You draw boxes. But instead of numbers, you write in dance moves. Like spin . . . bounce . . . or shake. Wherever it lands, that's your thing! If it lands on the question mark, do anything. That's "Hip Hop Scotch" in Houston.
VOICE-OVER Everyday is game day, so get out there and go play.
VOICEOVER For more cool games, log on to VERB at VERBnow.com. VERB. It's what you do. Made Possible by the U.S. Department of Health and Human Services and the Centers for Disease Control and Prevention.

##### Paid media advertising

The primary vehicle for reaching into the home is paid advertising in general market and ethnic media channels. VERB commercials air on age-appropriate television and radio channels such as Cartoon Network, Nickelodeon, The WB, ABC Saturday Morning Disney (including Radio Disney), Telemundo, and BET. Print advertising is placed in youth publications such as *Sports Illustrated for Kids, TIME for Kids, Teen People,* and *xSeventeen*. Examples of parent publications include *Family Circle, Parent Magazine, Ebony,* and *Indian Country Today*. Spanish and Asian in-language advertising and advertorials appear in publications such as *Korea Times, World Journal*, and *Los Padres*. (An inventory of current VERB advertising is available from http://www.cdc.gov/youthcampaign/advertising/index.htm.)

##### Added-value opportunities

In addition to paid media placements, the campaign negotiates added-value opportunities from media partners. VERB’s media partners donate their talent and properties or placements to help promote VERB’s physical activity messages to tweens. For example, media partners have produced VERB PSAs using their television talent, such as the stars of The WB’s *Gilmore Girls* and *7^th^ Heaven* and properties such as Disney’s *Kim Possible* and Cartoon Network’s *Courage the Cowardly Dog*. The PSAs are aired in prime time during these shows. For parents, The WB produced VERB PSAs featuring Reba McEntire and CBS produced VERB PSAs featuring Deion Sanders. Other examples include VERB sponsorship of Nickelodeon’s *Wild and Crazy Kids Show, Sports Illustrated for Kids’ Road Show*, and *Teen People’s Break for the Beach*. These added-value PSAs and sponsorships increase the “cool factor” and marketing reach of the VERB campaign among tweens.

##### Activity promotions

Several times a year, VERB features promotions that invite community-based organizations and schools throughout the United States to participate. For example, in 2003, VERB proclaimed the day of the summer solstice (the longest day of the year) as the “Longest Day of Play” and created a promotion with Radio Disney to motivate tweens to be active all day long. During fall 2003, when the clocks were turned back one hour from daylight saving to standard time, VERB featured its “Extra Hour for Extra Action” (EHEA) promotion, which included a kit of innovative and fun VERB materials for teachers and youth-serving organizations to use in activating tweens. Participating EHEA schools and organizations were eligible to apply for a small grant to support physical activity at the end of the three-week promotion.

**Figure:**
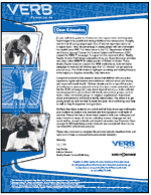
Download the Weekly Reader (PDF 477K) VERB™ — A Social Marketing Campaign to Increase Physical Activity Among Youth

**Figure:**
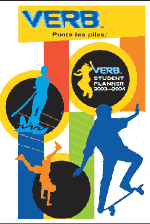
VERB Student Planner 2003–2004 Download the current Student Planner (PDF 16mb). This planner is also available from VERB in HTML format.

##### Schools

School is a natural venue for reaching tweens; it also gives youth a prime opportunity to discover their interests and develop skills. For example, working with youth publications like *Weekly Reader* and *TIME for Kids*, VERB distributes custom-developed materials to middle schools throughout the country. Primedia’s *Channel One* allows VERB advertising to reach tweens through schools. In-school vehicles include book covers, day planners, and customized lesson plans that incorporate physical activity into the classroom and encourage tweens to try many different VERBs.

##### Community-based events and grassroots marketing

 VERB participated in existing community events, including cultural festivals such as the Harvest Moon Festival (Los Angeles, Calif), Calle Ocho (Miami, Fla), and the Gathering of Nations Pow Wow (Albuquerque, NM). At these grassroots community events, VERB hosted an “activity zone,” a dedicated space for tweens to try out different activities such as kicking a soccer ball, dancing, performing martial arts, or other activities. Another community-based tactic is the use of “street teams,” teams of five to eight college-aged men and women hired to engage tweens in being physically active at events and tween hangouts, including malls, parks, and community centers. Street teams create buzz about VERB and build affinity for the brand as tweens tell their friends and siblings about their fun experiences and show off their VERB premiums. The street teams distribute VERB-branded premiums to tweens, such as foot bags, T-shirts, temporary tattoos, and Frisbee disks.

##### Contests and sweepstakes

To increase the value of the product and reward tweens for being active, many media partners sponsor VERB contests and sweepstakes. For example, *Channel One* sponsored a pedometer-based middle-school competition, *Make Every Move Count*. The schools that accumulated the most steps won an "Action Pack" of physical activity equipment and materials to support their physical activity programs. In addition, *YM (Your Magazine)* featured the VERB *Move It to Groove It* contest, where tween contestants competed to win a video dance party for their entire school.

##### Public relations

VERB continuously communicates with the news media, stakeholders, and partner organizations to offer information on the importance of youth physical activity to parents and other influencers and to spotlight current campaign activities, such as events and promotions. The campaign maintains good relationships with key members of the tween/teen and parent news media to keep them current on the campaign and to serve as resources for information about youth physical activity, childhood overweight, and related topics. News media materials are tailored to meet specific needs, media tours are conducted in key markets, and special news media coverage is arranged when appropriate.

##### Community partnerships

In the first year of VERB, the campaign developed local partnerships in the nine cities that received extra marketing activities to bring the VERB brand to life and to establish a foundation of support in those locations. Now, the goal of VERB is to recruit organizations across the country to become site partners (organizations that can provide opportunities for tweens to be physically active) or outreach partners (organizations that can reach parents or influence the environment to support tweens' participation in physical activity). The campaign is actively reaching out to organizations that have a network of affiliates or chapters across the country and also is contacting regional, state, and local organizations. (More information on VERB partnerships is available from http://www.cdc.gov/youthcampaign/partners/index.htm)

**Figure:**
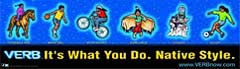
VERB poster "It's What You Do. Native Style."

**Figure:**
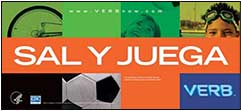
VERB poster "Sal y Juega."

##### Corporate partnerships

VERB also is seeking partnerships with corporations to extend the reach and appeal of the campaign to tweens. For example, VERB is successfully negotiating partnerships with professional sports leagues for its ProVERB initiative; those who have made commitments include the National Football League, National Hockey League, Major League Soccer, and Women’s Tennis Association. These sports leagues will provide content for the VERBnow.com Web site for tweens, donate athlete-signed merchandise for prizes, and provide opportunities for VERB sponsorship of their grassroots sports clinics.

##### Web sites

In partnership with AOL, VERB has created http://www.VERBnow.com, a Web site designed exclusively for tweens that includes the VERB Recorder, where tweens can report their participation in physical activity and become eligible to win prizes for being active. A parent site, http://www.VERBparents.com, includes in-language pages (Spanish, Chinese, Korean, and Vietnamese) in partnership with ethnic media partners. In addition, http://www.cdc.gov/VERB was created for partners and stakeholders to access information about the VERB campaign and to view advertising.

## Summary

The VERB campaign is a public health and marketing partnership based on the social marketing principles of product, price, place, and promotion. It brings together a diverse array of public health, marketing, and community experts to engage tweens in being physically active every day by playing, having fun, and trying new VERBs, or new ways to be physically active. A lifestyle or behavior change such as increasing physical activity is difficult to achieve and even more difficult to sustain. One can speculate, however, that success in changing behavior among tweens is more likely to be achieved when the following conditions are met: consumers have an in-depth understanding of the product and price associated with it, they have easy access to appropriate places where they can perform the behavior in everyday life, and product promotion portrays benefits in a positive, appealing fashion and reaches audiences through channels they value.

Rigorous multiyear evaluation of the VERB campaign will determine its effectiveness in motivating tweens to be more active. To ensure objectivity, the CDC has retained an evaluation contractor. The outcome evaluation is designed as a nationally representative longitudinal study of more than 6000 tweens and their parents across the country, half of whom are from the six high-dose communities. A baseline survey was conducted before the campaign's launch in June 2002. Two follow-up surveys, one conducted in 2003 and one to be completed in 2004, will measure the effectiveness of the campaign. The evaluation methodology, a longitudinal dose-response analysis, controls for numerous baseline factors and allows evaluators to measure changes in physical activity specifically attributable to the VERB campaign.

## Footnotes

1 Gross rating points is a measure of estimated exposure to advertising. Reach is defined as the proportion of the target audience that has an opportunity to be exposed to the ad. Frequency is the number of times an average target audience member is estimated to have an opportunity to view the advertisement in a given time period, usually weekly or monthly. Multiplying reach times frequency produces the GRP measure. Because GRPs are the product of reach and frequency estimates, a GRP estimate reflects many different possible exposure patterns. For example, if 10% of a population could be reached five times in a week, the weekly GRPs for that advertisement would be 50 (10 x 5); if 50% of the population could be reached once in a week, the GRPs would also be 50 (50 x 1).
