# Supplementary material for: VERB™ — A Social Marketing Campaign to Increase Physical Activity Among Youth
Source: Prev Chronic Dis. 2004 Jun 15;1(3):A10. (PMC1253475)
Supplement: Supplementary file 1 [file 04_0043_01.pdf]

VERB™
It's what you do.

Communicating VERB™: Keeping it Fun for Children

Tweens say, "VERB is cool," and "VERB is about getting active." The power of a cool and exciting VERB brand is that once the brand is strong and meaningful, it becomes A MESSAGE FOR LIFE.

Organizations can capitalize on VERB's popularity to offer opportunities for tweens to play and be physically active every day. Here are tips for keeping VERB motivating, fun, exciting, and a campaign for kids—not grown-ups. Let's keep VERB fun for tweens!

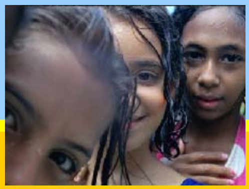

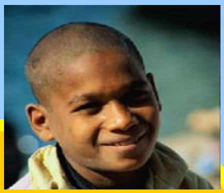

Communicating about VERB with tweens

- VERB is "for tweens, by tweens," so speak to them in "kid's language," include only kid relevant information, and keep VERB all about having fun and being with friends.
- VERB features positive, "can do," messages so don't diminish the brand's appeal with adult delivered, "must do," or "don't do," messages.
- VERB encourages trial and praise—"Try it to see if you like it." "You had so much fun today." "Dad would be so proud to know you….."
- VERB is solely about physical activity so don't confuse the brand with important healthy eating, obesity, anti-smoking, anti-drug, or other health messages.
- VERB can be in schools but VERB is not about health lessons, homework, or grades so keep it activity-focused, inspiring and fun.

Communicating about VERB with adults

VERB messages to adults must be kept separate from communications to tweens to maintain a cool VERB brand for tweens. For adults only, it is appropriate to

- Highlight the health concerns and benefits of youth physical activity.
- Offer ideas for encouraging, supporting, and recognizing tweens' trial and participation in physical activities.

VERB Brand Logo Usage: www.cdc.gov/youthcampaign/working_together/materials_logo_usage.htm

VERB™ is a trademark of the U.S. Department of Health and Human Services' Centers for Disease Control and Prevention. ©2003 Centers for Disease Control and Prevention. All rights reserved.

CDC
