# Supplementary material for: VERB™ — A Social Marketing Campaign to Increase Physical Activity Among Youth
Source: Prev Chronic Dis. 2004 Jun 15;1(3):A10. (PMC1253475)
Supplement: Supplementary file 5 [file 04_0043_02.pdf]

VERB™
It's what you do.

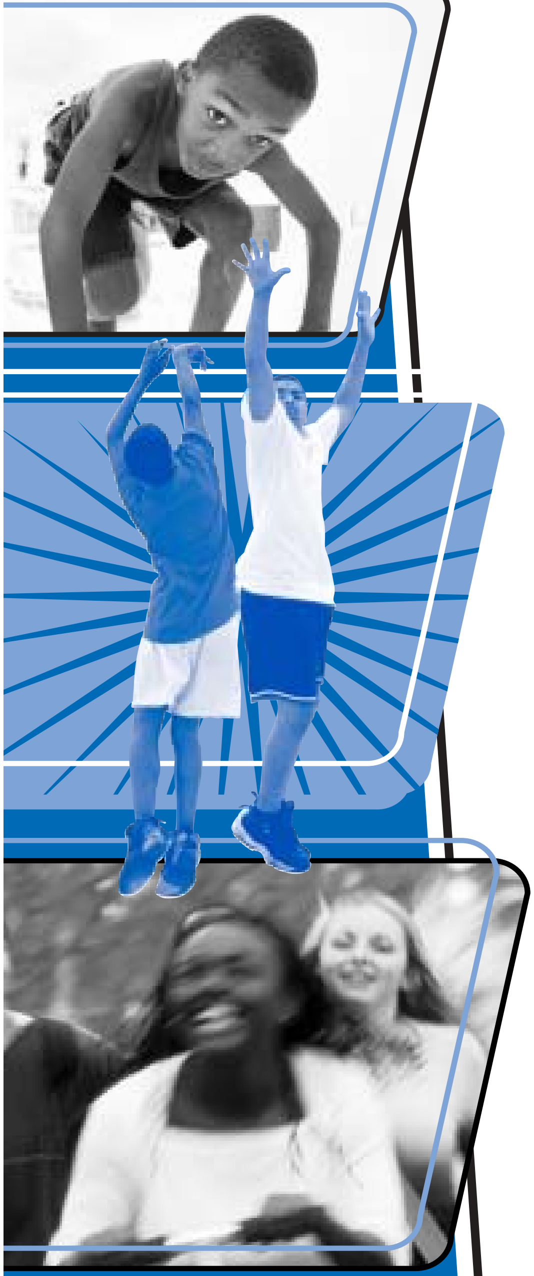

Dear Educator,

As you well know, patterns of behaviors that impact short- and long-term health begin to be established during childhood and adolescence. Roughly one-third of all young people ages 12 to 21 are not vigorously active on a regular basis.[1] And, the percentage of young people who are overweight has tripled since 1980.[2] For these reasons, the U.S. Department of Health and Human Services' Centers for Disease Control and Prevention (CDC) created the **VERB.™** campaign. In support of this important effort, Weekly Reader Custom Publishing is pleased to bring you these classroom activities and video called **VERB.™ It's what you do. In School. In Action.** These Weekly Reader materials support the VERB multicultural, national media campaign to motivate *all* children ages 9 to 13 ("tweens") to get active in positive ways. The VERB initiative highlights enriching and healthy lifestyles, while helping to displace unhealthy, risky behaviors.

Compared to inactive kids, research shows that children who are active experience higher self-esteem and confidence, reduced stress and anxiety, and improved overall health.[3] Children should aim for at least 60 minutes of moderate-to-vigorous physical activity five days a week, preferably daily.[4] But the VERB campaign isn't only about physical activity — it also includes encouraging kids to get involved with family and positive groups, such as teams, clubs, community groups or religious organizations. It promotes social connectedness and positive family relationships. The intent is to get young people up and moving! To reach this goal, we are enlisting your help as well as help from parents and guardians.

We hope that these materials are useful and will help encourage and inspire your students to explore new interests and find enjoyment in all kinds of activities. Please feel free to share these materials with your colleagues and adapt them to a variety of courses, including science, language arts and social studies, as well as health and physical education (see Extension Ideas on page 3). Although these printed materials are copyrighted, they may be reproduced for educational purposes.

Please take a moment to complete the enclosed educator feedback form and reply card to help us prepare future educational materials.

Sincerely,

Katy Dobbs

Katy Dobbs
Editorial Director
Weekly Reader Custom Publishing

VERB™
It's what you do.

WEEKLY READER®

To print or order copies of a free brochure filled with ideas for integrating physical activity into classroom instruction, go to *http://www.cdc.gov/HealthyYouth/PhysicalActivity* or call *(888) 231-6405*.

[1] "Youth Risk Behavior Surveillance: United States, 2001." www.cdc.gov/mmwr/preview/mmwrhtml/ss5104a1.htm
[2] "Overweight Among U.S. Children and Adolescents." National Center for Health Statistics, 2002. www.cdc.gov/nchs/data/nhanes/databriefs/overwght.pdf
[3] "Guidelines for Schools and Community Programs to Promote Lifelong Physical Activity Among Young People." Centers for Disease Control and Prevention, 1997. www.cdc.gov/nccdphp/dash/healthtopics/physical_activity/guidelines
[4] "CDC's Guidelines for School and Community Programs: Promoting Lifelong Physical Activity." Centers for Disease Control and Prevention, 2000. www.cdc.gov/nccdphp/dash/00_pdf/phactaag.pdf

Target Audience

Students in grades 4 and 5

Program Objectives

- *To motivate students in grades 4 and 5 to get positively active in ways that are physical and done in organized groups.*
- *To promote healthy lifestyles among this age group and displace unhealthy, risky behaviors.*
- *To encourage tweens to try something new, find activities they enjoy doing and keep doing them.*

Teacher Objective

- *To convey to students and parents the benefits of being active: improved health; self-expression, aspirations for the future and fun; enhanced sense of well-being and social belonging; enhanced self-esteem and confidence.*

These materials address components of the National Education Standards for Level II:

Health

- ***Standard 7 —*** *Knows how to maintain and promote personal health.*

Physical Education

- ***Standard 3 —*** *Understands the benefits and costs associated with participation in physical activity.*
- ***Standard 4 —*** *Understands how to monitor and maintain a health-enhancing level of physical fitness.*
- ***Standard 5 —*** *Understands the social and personal responsibility associated with participation in physical activity.*

Program Components

- *Four reproducible student pages*
- *Four-page teacher's guide (including a reproducible letter for parents)*
- *A five-minute video,* Keep Moving
- *One sheet of 30 stickers*
- *A wall poster*
- *A teacher feedback form and reply card*

Getting Started

Show your class the five-minute video, *Keep Moving*, that shows kids how to be active throughout their day, have fun finding out what they like to do, and enjoy those things with their friends. Ask your students if they identify themselves with any of the kids seen in the video.

Prior to distributing the activity sheets to your students, discuss with them what kinds of activities they like to do. Take a tally to see which activities are the most popular. Ask students how often they do these activities, and with whom they do them.

It is important that all students participate, all ideas are welcome and students are encouraged to name more than sedentary behaviors like watching TV and playing video games.

Page One — VERB. Why you do it.

Background and Objective

This first student page focuses on the benefits of participating in physical activities such as sports and dance. Physical activities can be prosocial: in a club, group or on a team. This first lesson serves as a motivator for kids to get active. Some reasons for them to be active are:

- *it is fun*
- *they can spend time with friends, family and neighbors*
- *they might discover a hidden talent or skill, or find something that they really love to do*
- *activities are a way to express themselves and communicate what they like to do*

Activity

Review with your class the reasons for getting active. Brainstorm what they do that is active and fun, who they do it with, what new things they have discovered that they like doing and how these things express who they are and what interests they have.

**Note: Competitive activities aren't for everyone — as a matter of fact, competition can take the fun out of being active. Try to encourage a full range of activities so all children can have fun.*

Encourage your students to get active — get their bodies and their minds moving. Children should be spending at least 60 minutes a day, at least five days a week, participating in a variety of moderate-to-vigorous physical activity individually, with friends or in an organized group. They should also consider participating in organized (or pro-social) activities such as painting, volunteering, playing in a marching band or singing. Organized (or prosocial) activities will help them build alliances with family, organized peer groups and conventional institutions, and will help to displace unhealthy, risky behavior.

**Note: Be supportive of your students' efforts to get moving. If they find enjoyment from being active, it is more likely that they will incorporate activity into their lifestyle for the long-term.*

Page Two — What's your VERB?

Background and Objective

This page helps students develop a plan to discuss their VERBs. It entails

- *setting a goal*
- *doing the activity*
- *keeping track of when they are active and the activities they try*
- *mixing it up to keep things new and exciting*
- *rewarding themselves for their efforts*

Activity

After discussing the page as a class, talk about each student's plan for getting active. Ask your students their main reasons for being active. How do they plan on reaching their goals? For example, if their goal is to do more things with their peers, they might join a local soccer team or take an after-school dance class.

Talk about the ideas for getting active found at the bottom of the activity page. Remind students that being active and finding their VERB doesn't necessarily mean joining a sports team or paying to use an ice skating rink. Being active is about getting up and doing something fun — it can be as simple as volunteering, helping to clean up a park, taking a walk or jog with a parent or dancing in the living room with a friend.

Divide your students into groups and give each group some equipment (i.e., a basketball and 10 cones, a Frisbee® and a hula hoop, a football and two jump ropes, etc.). Ask students to develop a game using all of their equipment. Have groups demonstrate their games for the class.

****Note: Students with disabilities can also be active. Recognize their physical abilities and be creative. Visit www.americasathletes.org or www.dsusa.org for more information.***

Some Resources

Student Sites

www.VERBnow.com
www.bam.gov
www.kidnetic.com
www.seventeen.com/VERB
www.cdc.gov/powerfulbones

Parent Sites

www.VERBparents.com
www.healthykids.com
www.fitfamilyfitkids.com
www.walkingschoolbus.org
www.ymca.net
www.bgca.org

Teacher and Professional Sites

www.cdc.gov/youthcampaign
www.bam.gov/teachers
www.fitness.gov
www.cdc.gov/nccdphp/dash
www.cdc.gov/nccdphp/dnpa
www.americasathletes.org
www.dsusa.org
www.pecentral.com
www.pepgrant.info/newpe.htm
www.aka.org.au

Page Three VERB. Where you do it.

Background and Objective

This page asks students to find information about where they can do the VERBs they like to do.

Activity

Students should look for community events and local organizations, including team sports, volunteer organizations, religious groups and after-school programs in their neighborhood. Give them time in class to find this information. Provide students access to conduct this research, such as the Internet, phone books, local newspapers and the library.

After students have compiled their research, discuss the information as a class so that as many local organizations and facilities as possible are talked about and explored.

**Note: Consider inviting a representative from a local organization, such as Boys & Girls Clubs, YMCA, or Parks & Recreation, to talk to the class about activity options, and/or demonstrate activities in which students showed an interest.*

Page Four VERB. It's what I do.

Background and Objective

This activity page will give your students the opportunity to think about the things they like to do and write about them.

Activity

Ask your students to list activities they like to do, new things that they've tried recently, new things they'd like to try and five active things that they've done this week. This activity page can serve as a template that can be used multiple times, so the students are constantly encouraged to try new things and keep active throughout the week.

You can also have students make their own VERB poster. Ask them to cut out magazine and newspaper clippings of their favorite ways to get active. Tell them to use verbs, or action words, associated with those activities to create a poster encouraging other people to get active. Display the posters in the hallways, gym and cafeteria.

Ask students to keep a journal of their activities. Hold weekly discussions on how many times a week your students are active, what new positive activities they are trying, where they are going to be active, etc. When a new activity or location is mentioned, ask that student to write it on a large index card and then decorate the card. Post these on a bulletin board as a reminder of all the possible activities that are out there for them to try.

Stickers

Make a class chart, or have each student make their own chart, to keep track of how many times a week each student is active. When they are active twice a week, give each student a VERB sticker to keep them motivated. Other rewards for being active can include:

- *Select from one of the activities brainstormed in Activity Two to try as a class on the playground.*
- *Play a card game or board game after students are done with their schoolwork.*
- *Present the class' favorite activity to a younger grade, and tell them why it's fun to get moving.*
- *As a group or class, organize an activity at your school's Field Day or Wellness Day.*

Parent Letter

On the back of the teacher's guide there is a reproducible letter for you to share with your students' parents and guardians. It is very important to convey to parents the importance of activity in their child's life, as well as the need for their support and encouragement. At the same time, we want to ensure that activity remains fun for kids. Therefore, we request that you distribute these letters to parents and guardians directly by mailing the letter home, sending it in a PTA distribution or with report cards or saving it for parent-teacher conferences.

To encourage parents to engage their children in positive activities, try these ideas.

- *Suggest that neighboring parents take turns supervising their children as they play together after school. This helps to keep the kids in a safe environment.*
- *Encourage the PTA to form a parent/guardian committee that is responsible for organizing group activities, such as a "walk to the park" night, chalk fest on the pavement, softball game or roller blading in the school parking lot. Work with your school's principal so that school property can be open beyond school hours to be available for activities and gatherings organized by this committee.*
- *Encourage parents, when feasible, to implement a "Walking School Bus" where parents take turns accompanying children walking to school and "picking up" others as they go. For more information, visit www.walkingschoolbus.org.*

Extension Ideas Curricula Links

Teachers and schools can also incorporate activity into learning.

- *Schedule regular recess and PE classes.*
- *Incorporate lessons from New PE, making physical education appropriate for everyone. For more information, visit* www.pepgrant.info/newpe.htm.
- *Do not use physical activity as a punishment (doing laps for being late).*
- *Do not withhold PE or recess as a punishment.*

Look for ideas to integrate activity and movement into your everyday lesson plans. It makes learning more fun, and will grab students' attention. Try these fun ideas to get you started. They can be adapted across multiple areas of the curricula.

***Go Fly A Kite* —** Teach your students about gravity, aerodynamics and wind by making and flying a kite. Visit the Australian Kite Association at *www.aka.org.au* for more information on lesson links and instructions.

***Energize Yourself* —** Convey the difference between Potential Energy and Kinetic Energy by giving students directives, such as march in place, take five steps forward, etc.

***VERBstorm* —** Give students, or groups of students, a verb such as twist, run, fly or groove, and ask them to list and demonstrate all of the activities and games that come to mind.

***VERBs I Do* —** Ask each student to write his or her first name vertically down the page. For each letter, ask them to write a verb that relates to them.

***Geography Movement* —** Give each group of students a region of the United States. For each state in their region, they should list the kinds of activities that can be done given the state's topography, climate, location, etc. As a class, discuss what the students came up with. For example: in how many states can you ski? go swimming in the ocean? take a dip in a lake?

***Freeze Facts* —** Make index cards with questions and answers to any topic you are studying. Now, play freeze tag outside or in the gym. Start by asking three-quarters of your class to skip, hop, jog or leap around. The other quarter (wearing different colored shapes taped to their shirts for distinction) tags them frozen. In order to be released, they must answer a question asked by any tagger correctly. Switch the taggers every couple of minutes to give everyone a turn.[5]

***Media Literacy* —** Have children count how many media messages (TV, radio, billboards, magazines, etc.) that they see in one day encouraging inactivity (promoting video games, marathon blocks of a TV show, etc.) compared to how many they see encouraging activity.

©2003 U.S. Department of Health and Human Services' Centers for Disease Control and Prevention. Created by Weekly Reader Corporation.

[5] Adapted from an activity on PE Central, www.pecentral.com.

Dear Parents and Guardians,

Many adults and children alike can benefit from regular physical activity. How many hours a week does your child spend in front of the TV or computer screen? The answer is probably *too much*. The U.S. Department of Health and Human Services' Centers for Disease Control and Prevention (CDC) has created the VERB.™ campaign to encourage children ages 9–13 to get active in an exciting and safe way. It is also about getting involved with family and organizations like clubs, community groups and religious organizations.

Your child has been working on an in-school activity called VERB.™ It's what you do. In School. In Action. as part of the VERB.™ It's what you do. media campaign. Developed by Weekly Reader Custom Publishing in support of VERB, it encourages your child to discover new activities and new ways to have fun. By fitting positive activity into your child's life you may help him or her stay out of unsafe or troublesome situations. You will also bring a special bond and element of fun to your family.

Children who are active often have higher self-esteem and confidence.[1] This can help them as they grow older and start to make important decisions on their own. By being active, children also have better overall health and have less stress and anxiety.[2] Children should try to be physically active for at least 60 minutes a day, five days a week, preferably every day.[3]

With your help and enthusiasm, your child and your family can become more active and healthier. Some ways to add positive activities to your child's life include:

- Talk to your child about activities that he or she finds interesting. Help him or her select safe, enjoyable activities.
- Encourage children to be active with their friends, and to play outside in safe and supervised places such as playgrounds and parks.
- Balance screen time (TV, video games, recreational computer use) with play time. Set limits on the amount of time your child spends watching television and playing video or computer games. (The American Academy of Pediatrics recommends no more than an hour or two a day spent watching TV.)
- Make a schedule with neighboring parents to take turns watching the children play together after school and on weekends. This helps keep your children in a safe environment.
- When possible, create a "Walking School Bus" where parents take turns walking children to school and "picking up" others as they go. For more information, visit www.walkingschoolbus.org.

To print or order copies of a free brochure filled with physical activity ideas for your children and family, go to *http://www.cdc.gov/HealthyYouth/PhysicalActivity* or call *(888) 231-6405*.

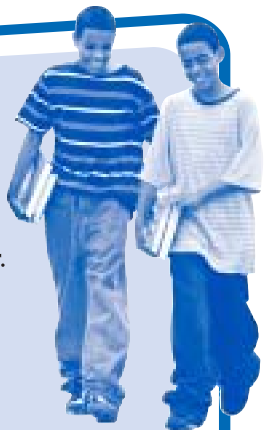

Try these ideas to get your family moving:

- Walk to the park or walk around the zoo.
- Roller skate or roller blade on Saturday mornings.
- Go for a bike ride.
- Play basketball, a game of catch, badminton, Frisbee®, stickball, volleyball or soccer.
- Go bowling, hiking or swimming.
- Volunteer together.
- If you've wanted to take a karate or dance class, rent a tape and try it out together as a family.

For more fun activity ideas and tips, check out these Web sites:

- www.VERBparents.com — Provides parents with fun activity ideas and tips.
- www.fitfamilyfitkids.com — Suggests strategies for improving your family's physical activity and nutrition habits.
- www.healthykids.com — Offers health information, fun games and activities and more.

Don't forget to check out the Web for community groups, parks and recreation, and religious groups for activities and ideas. A few Web sites that may be helpful are the YMCA (www.ymca.net) and the Boys and Girls Clubs of America (www.bgca.org).

[1] "Guidelines for Schools and Community Programs to Promote Lifelong Physical Activity Among Young People." Centers for Disease Control and Prevention, 1997. www.cdc.gov/nccdphp/dash/healthtopics/physical_activity/guidelines

[2] "Guidelines for Schools and Community Programs to Promote Lifelong Physical Activity Among Young People." Centers for Disease Control and Prevention, 1997. www.cdc.gov/nccdphp/dash/healthtopics/physical_activity/guidelines

[3] "CDC's Guidelines for School and Community Programs: Promoting Lifelong Physical Activity." Centers for Disease Control and Prevention, 2000. www.cdc.gov/nccdphp/dash/00_pdf/phactaag.pdf

©2003 U.S. Department of Health and Human Services' Centers for Disease Control and Prevention. Created by Weekly Reader Corporation.
